# Shortening the decade‐long gap between adult and paediatric drug formulations: a new framework based on the HIV experience in low‐ and middle‐income countries

**DOI:** 10.1002/jia2.25049

**Published:** 2018-02-27

**Authors:** Martina Penazzato, Linda Lewis, Melynda Watkins, Vineet Prabhu, Fernando Pascual, Martin Auton, Wesley Kreft, Sébastien Morin, Marissa Vicari, Janice Lee, David Jamieson, George K Siberry

**Affiliations:** ^1^ World Health Organization Geneva Switzerland; ^2^ Clinton Health Access Initiative Boston MA USA; ^3^ Medicines Patent Pool Geneva Switzerland; ^4^ The Global Fund to Fight AIDS, Tuberculosis and Malaria Geneva Switzerland; ^5^ Partnership for Supply Chain Management Amsterdam the Netherlands; ^6^ International AIDS Society Geneva Switzerland; ^7^ Drugs for Neglected Diseases initiative Geneva Switzerland; ^8^ Partnership for Supply Chain Management Washington DC USA; ^9^ Office of the U.S. Global AIDS Coordinator U.S. Department of State Washington DC USA

**Keywords:** paediatric drugs, drug development, drug formulations, regulatory approval, Global Accelerator for Paediatric Formulations, HIV, tuberculosis, viral hepatitis

## Abstract

**Introduction:**

Despite the coordinated efforts by several stakeholders to speed up access to HIV treatment for children, development of optimal paediatric formulations still lags 8 to 10 years behind that of adults, due mainly to lack of market incentives and technical complexities in manufacturing. The small and fragmented paediatric market also hinders launch and uptake of new formulations. Moreover, the problems affecting HIV similarly affect other disease areas where development and introduction of optimal paediatric formulations is even slower. Therefore, accelerating processes for developing and commercializing optimal paediatric drug formulations for HIV and other disease areas is urgently needed.

**Discussion:**

The Global Accelerator for Paediatric Formulations (GAP‐f) is an innovative collaborative model that will accelerate availability of optimized treatment options for infectious diseases, such as HIV, tuberculosis and viral hepatitis, affecting children in low‐ and middle‐income countries (LMICs). It builds on the HIV experience and existing efforts in paediatric drug development, formalizing collaboration between normative bodies, research networks, regulatory agencies, industry, supply and procurement organizations and funding bodies. Upstream, the GAP‐f will coordinate technical support to companies to design and study optimal paediatric formulations, harmonize efforts with regulators and incentivize manufacturers to conduct formulation development. Downstream, the GAP‐f will reinforce coordinated procurement and communication with suppliers. The GAP‐f will be implemented in a three‐stage process: (1) development of a strategic framework and promotion of key regulatory efficiencies; (2) testing of feasibility and results, building on the work of existing platforms such as the Paediatric HIV Treatment Initiative (PHTI) including innovative approaches to incentivize generic development and (3) launch as a fully functioning structure.

**Conclusions:**

GAP‐f is a key partnership example enhancing North‐South and international cooperation on and access to science and technology and capacity building, responding to Sustainable Development Goal (SDG) 17.6 (technology) and 17.9. (capacity‐building). By promoting access to the most needed paediatric formulations for HIV and high‐burden infectious diseases in low‐and middle‐income countries, GAP‐f will support achievement of SDG 3.2 (infant mortality), 3.3 (end of AIDS and combat other communicable diseases) and 3.8 (access to essential medicines), and be an essential component of meeting the global Start Free, Stay Free, AIDS Free super‐fast‐track targets.

## 1 Introduction

Prompt treatment of people living with HIV (PLHIV) with appropriate antiretroviral drugs (ARVs) saves lives and improves health, but the 43% ARV treatment (ART) coverage of children living with HIV (CLHIV, <15 years old) continues to lag behind adult coverage 1, and many CLHIV in low‐ and middle‐income countries (LMIC) still do not receive optimal paediatric formulations. In an era that has seen the major public health achievement of 18.2 million people accessing ARVs worldwide in 2016 1 and new fast track targets to end AIDS by 2030 2, drug development for children surprisingly still lags 8 to 10 years behind that of adults 3. There are many demographic, structural, regulatory, technical and economic challenges slowing drug development for children. The 2.1 million CLHIV globally make up less than 10% of all PLHIV, but require combinations, strengths and formulations of ARVs that vary by age and weight. The result is a fragmented, low‐volume market that typical economic incentives will not address, making it difficult for generic drug manufacturers to engage 4, even when there are no intellectual property barriers. Declining numbers of new infections in children coupled with technical challenges in making child‐friendly formulations and the need to investigate safety and dosing across the paediatric spectrum of ages and weights contribute to delays in testing of new ARVs in children, despite regulatory incentives and requirements for paediatric development plans for new drugs by innovator companies 5. Individual country regulatory approvals, national HIV treatment policies and supply chain management strategies can lead to further delays in uptake into treatment programmes. These challenges are not unique to HIV; similar barriers have been encountered in developing optimal formulations for the treatment of tuberculosis and viral hepatitis in children. Small markets, particularly for multi‐drug resistant (MDR) tuberculosis, and unclear treatment recommendations for children, such as for hepatitis B and hepatitis C, have resulted in very slow progress and lack of access to paediatric formulations.

Stakeholders working in the area of paediatric HIV have come together in an unprecedented effort of cross‐sectoral collaboration to develop and articulate solutions to these challenges. The work stream has been unified by linking core activities of policymakers, research networks, regulatory agencies, manufacturers, supply and procurement organizations and funding bodies with the aim of ensuring accelerated development and uptake of optimal ARVs for children. A proposal now stands to take this to the next level with a more formalized, sustainable, coordinated framework aiming to accelerate processes for development and uptake of prioritized paediatric drug formulations for use in LMICs and globally by 2020: the Global Accelerator for Paediatric Formulations (GAP‐f). GAP‐f will accelerate both upstream and downstream processes for developing paediatric drug formulations for HIV and for other disease areas, like hepatitis C and tuberculosis that face the similar challenges of young target populations, high burden in LMICs, small‐volume markets and lack of market incentives for development and manufacturing.

GAP‐f will make an important contribution to ensure success of Sustainable Development Goal (SDG) 3: ensure healthy lives for all and promote wellbeing. Ensuring access to the most needed paediatric formulations for HIV and other high‐burden infectious diseases in LMICs directly responds to SDG 3.8 (access to essential medicines, indicator 3.8.1), as development and uptake of the most needed paediatric formulations will overcome the principal barrier to access for these populations. It will help to reduce HIV‐related infant and child mortality in support of SDG 3.2 (indicators 3.2.1, Under‐five mortality rate and 3.2.2 Neonatal mortality rate) and is necessary to help end AIDS and combat other communicable diseases (SDG 3.3, indicators 3.3.1, 3.3.2 and 3.3.3). Finally, it is an essential component of meeting the global Start Free, Stay Free, AIDS Free super‐fast‐track targets for paediatric HIV 6.

GAP‐f exemplifies partnership and collaboration that enhance North‐South and international cooperation on and access to science and technology, and thus it directly responds to SDGs 17.6 (technology, indicator 17.6.1) and 17.9 (capacity‐building, indicator 17.9.1).

## 2 Discussion

### 2.1 What is GAP‐f?

Since 2013, under the coordination of the World Health Organization (WHO), cross‐sectoral collaboration in paediatric HIV has increased among key stakeholders addressing medium‐ and long‐term prioritization of most needed paediatric formulations for development. This has been accomplished through several ongoing initiatives:

1. The Paediatric ARV Drug Optimization (PADO) group sets priorities for development of new ARV drug formulation for children.
2. The Paediatric ARV Working Group (PAWG) provides technical guidance on weight‐band dosing and pharmacokinetic and acceptability studies of ARV drugs in children.
3. The Interagency Task Team on Prevention of HIV Transmission in Pregnant Women, Mothers and their Children (IATT) develops a Paediatric ARV Formulary of existing drug formulations needed from manufacturers to enable optimal treatment of children.
4. The Paediatric ARV Procurement Working Group (PAPWG) coordinates procurement of paediatric ARVs for approximately 70 LMIC programmes.

In 2014, UNITAID, the Drugs for Neglected Diseases initiative (DNDi) and the Medicines Patent Pool (MPP), launched the Paediatric HIV Treatment Initiative (PHTI), to develop and deliver specific paediatric formulations; the Clinton Health Access Initiative (CHAI) joined the PHTI later. Later in 2014, partners came together to advance the paediatric HIV agenda under the umbrella of the Global Pediatric Antiretroviral Commitment‐to‐Action (CTA). Several broad consultations held in 2016 7, 8, 9 explored mechanisms to advance paediatric formulation development and introduction. In parallel, two meetings organized under the leadership of the Holy See 10 generated high‐level support to facilitate closer collaboration between the private sector and relevant stakeholders. These efforts to support paediatric formulation development and uptake are essential elements of the AIDS Free agenda of the Start Free, Stay Free, AIDS Free super‐fast‐track framework for ending AIDS in children, adolescents and young women by 2020, launched by UNAIDS and PEPFAR in 2016 11.

**Figure 1:**
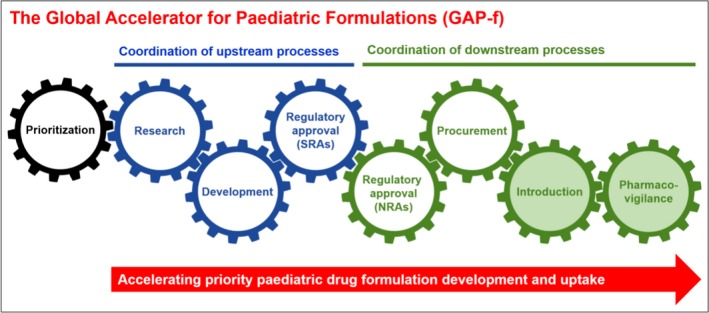
The Global Accelerator for Paediatric Formulations (GAP‐f). The GAP‐f formalizes collaboration across sectors to ensure accelerated development and uptake of the most needed drugs and formulations for children. SRAs, stringent regulatory authorities; NRAs, national regulatory authorities (in high‐burden countries).

The GAP‐f brings together these efforts through establishment of a more formalized mechanism with collaboration upstream (clinical and formulation development by innovators and generics; stringent drug regulatory authority filing and approval processes; optimized paediatric product testing and generic manufacturing) and downstream (country‐level drug regulatory approval; national treatment policy; supply chain management; programme sensitization; market uptake and incentives). The GAP‐f will streamline efforts currently underway, integrating and consolidating all stakeholders invested in different steps of the pathway of paediatric drug prioritization, development, manufacture and uptake into a coherent, single‐framework mechanism (see Figure 1).

### 2.2 Finding efficiencies upstream

**Figure 2:**
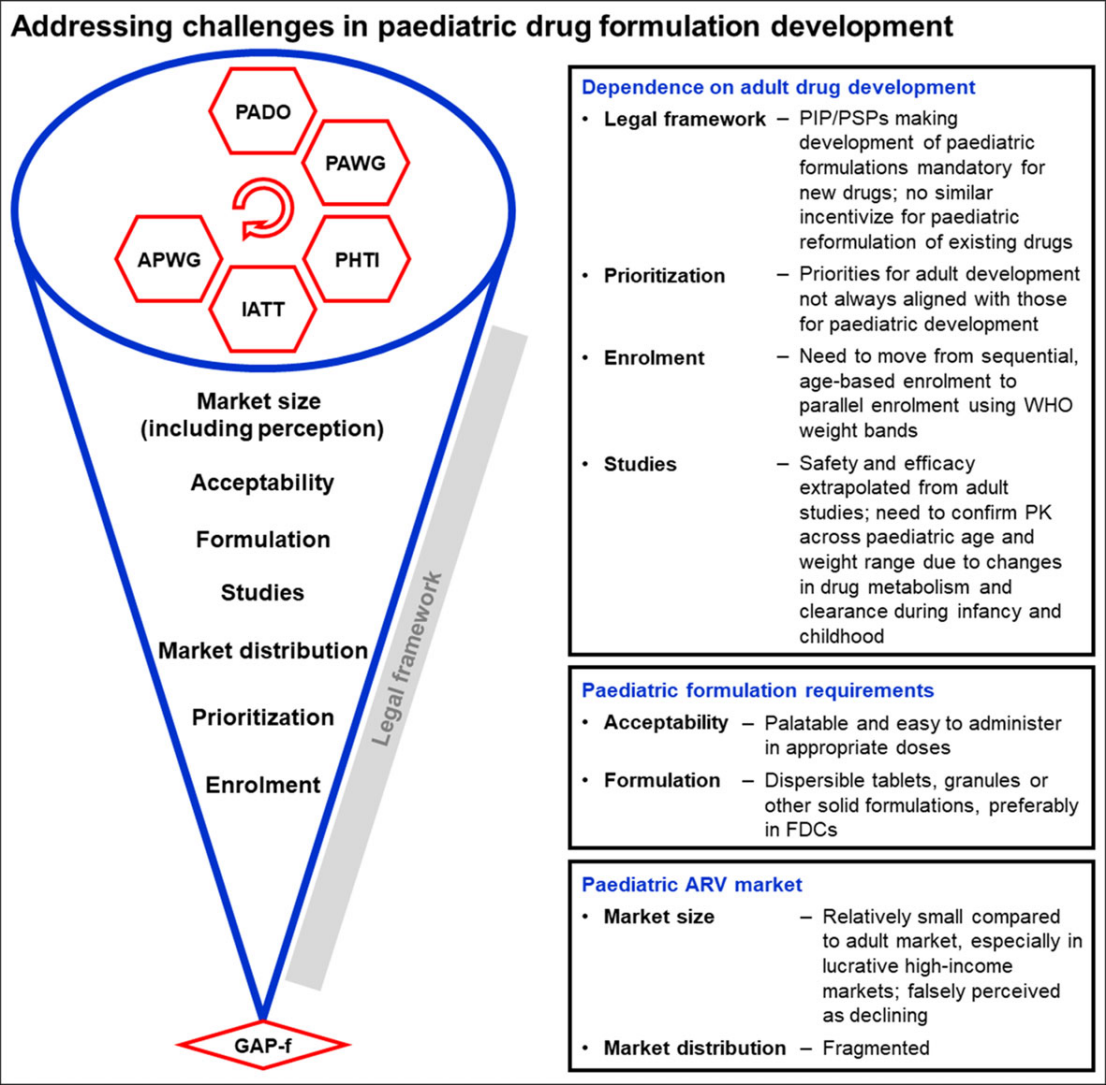
The GAP‐f represents an opportunity to address challenges in paediatric drug formulation development. Challenges are grouped around three areas: dependence on adult drug development, paediatric formulation requirements and paediatric ARV market. Progress to‐date in addressing these challenges is depicted along a funnel originating from precursor mechanisms and leading up to the GAP‐f collaborative model. Legal framework challenges are placed outside of the funnel because of the limited influence of the GAP‐f to directly address these. PK, pharmacokinetic; GAP‐f, Global Accelerator for Paediatric Formulations; ARV, antiretroviral.

Currently, the development of paediatric products is closely dependent on development of products for adults, although it presents additional challenges (see Figure 2). Efficacy of most drugs (including ARVs) in children is extrapolated from results of clinical trials conducted in adults; direct studies of safety and dosing in children across the paediatric age and weight spectrum are then required. Through the submission of paediatric investigation plans [PIP, at the European Medicines Agency (EMA)] or paediatric study plans [PSP, at the United States Food and Drug Administration (US FDA)], innovator companies commit to generate supportive data in children required for authorization of a medicine for paediatric use. These plans, compulsory for all companies seeking marketing approval unless they obtain a waiver or a deferral, are submitted very early in drug development, and include non‐clinical and clinical study plans 12, 13. The execution of these plans starts after proof of concept of the adult product and represents a large investment from pharmaceutical companies.

Formulation development is also a critical element of paediatric drug development. Paediatric products must be age‐appropriate formulations for the intended age groups, palatable and easy to administer in appropriate doses. In the past, most paediatric formulations were oral liquids, which are difficult to store, may need refrigeration, entail more complex administration (with higher risk of dosing errors) and are more difficult for making fixed‐dose combination (FDC) products. However, WHO currently recommends dispersible tablets, granules or other solid formulations (that do not require whole pill swallowing), preferably in FDCs, to avoid the complexities linked to liquid administration 14. Currently, there are several key regimens recommended by WHO available in such child‐friendly formulations (e.g. ABC/3TC dispersible tablets, LPV/r pellets), but still more are needed. In addition, it is important to ensure that future products are developed following these recommendations.

Because the paediatric market for HIV and other infectious disease products is small, innovator companies that have previously developed and launched paediatric formulations imperfectly adapted for use in LMICs are unlikely to reformulate their products. In such cases, generic companies may be best placed to manufacture alternate formulations for existing products when patents expire or when the innovator companies grant voluntary licences (VL) permitting generic versions of drugs with remaining patent protection. Since 2010, the MPP has been striving to negotiate VL agreements with innovators of HIV, hepatitis C and tuberculosis medicines 15. To date, all innovators have granted VLs to the MPP for all WHO‐recommended paediatric ARVs still under patent (except for darunavir, for which Janssen announced intent not to enforce patents in resource‐limited settings 16).

Regulatory approval of novel formulations, especially new FDC products for which component drugs are owned by different innovators, was previously out of the scope of stringent regulatory authorities (SRA). In the last decade, several initiatives have addressed some of these limitations mainly in the field of HIV. In 2006, as part of the President's Emergency Plan for AIDS Relief (PEPFAR), the US FDA identified a mechanism to grant tentative approval to ARV products intended for procurement in developing countries while maintaining patent protection within the US 17. EMA now gives to manufacturers, scientific opinion on the regulatory requirements for products intended for non‐EU markets through the Article 58 procedure. In addition, the WHO prequalification team assesses products and inspects manufacturing plants. As a result, several paediatric‐adapted formulations, including several dispersible FDCs for HIV, TB and malaria, that meet the high SRA standards for efficacy, safety and quality are available to children in LMICs.

**Figure 3:**
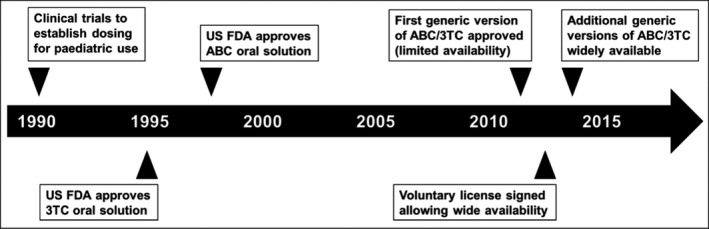
Timeline for ABC/3TC development. It took almost 15 years after ABC and 3TC were first approved for use in children until enough generic versions of child‐friendly formulations were produced to make these drugs widely available to children in LMIC. ABC, abacavir; 3TC, lamivudine; LMIC, low‐ and middle‐income countries.

A good illustration of the paediatric development process is the FDC containing abacavir (ABC) and lamivudine (3TC), key components of WHO‐recommended first‐line HIV treatment for children (see Figure 3). The innovator conducted clinical trials in the 1990s to establish the appropriate dose for children. The US FDA approved the oral solutions in 1995 (3TC) and 1998 (ABC). The first generic dispersible tablet containing a combination of the two products was approved in 2011 and was only available in countries where there was no patent restriction. Only in 2014, four years after WHO recommended the use of this combination as a preferred backbone in first‐line treatment for all children 18 were other generic versions made more widely available. Bilateral licensing agreements between the innovator and generic companies and VLs with the MPP for ABC expanded access to these formulations to 121 countries.

This example shows the need to accelerate the development of new products and formulations in HIV, and even more in other disease areas where progress has been slower. Newer generation ARVs and direct‐acting antivirals for hepatitis C infection, among others, offer the possibility of testing new models, such as the one proposed by the GAP‐f, to accelerate development of optimal formulations. Direct and early technical support by the PAWG can help define and accelerate paediatric development plans (PIP and PSP) and clinical studies. It is also important that innovator companies develop formulations and combinations appropriate for the global population ensuring intellectual property is not a barrier. Coordination between regulators to exchange information on paediatric development plans must occur regularly and early enough so plans can be aligned to product needs. Harmonized protocols for paediatric trials can optimize safety and efficacy data, and thus simplify development. Finally, incentivizing generic manufacturers to conduct formulation development can mitigate the negative effects of the small market size. The incentives could include technical assistance in the design of studies needed to obtain regulatory approval that rely on published literature or on data supporting a previous approval, as well as financial support to conduct such studies.

### 2.3 Increased coordination downstream

In‐country registration is often the bottle neck for rapid market introduction. More systematic use of existing (Collaborative Registration Procedure CRP for WHO‐prequalified products: http://apps.who.int/medicinedocs/en/d/Js21317en/) mechanisms to enable increasing reliance by NRAs on regulatory approval granted by SRAs is expected to significantly accelerate approval processes. These mechanisms would, in fact minimize the need to undertake additional local clinical studies when robust evidence already exists.

The impact of new products on patients’ lives relies as much on what happens downstream of development and regulatory approval as upstream. Careful procurement planning and clear communication between procurers, programmes and suppliers can ease the launch of a new paediatric formulation. In the absence of this, suppliers can be hesitant to take on inventory risk and commit production resources to the new product(s) until larger orders are received, increasing lead times, and risking loss of interest from programmes to adopt new product(s), or worse still, stock‐outs. Avoiding stock‐outs and delivering optimal products facilitates achievement of the SDG 3, including ending preventable deaths of newborns and children <5 years of age, and ending the AIDS epidemic by 2030.

The paediatric ARV market, despite growth over the last 10 years, remains relatively small and fragile. To address procurement and access challenges, the PAPWG was created to lead global collaboration and coordination among key partners, including procurement that promotes optimal products. This effort has succeeded in consolidating the number of different paediatric ARV products procured and increasing the share of paediatric products procured that correspond to WHO‐preferred products for children. Following success in the paediatric market, in 2016, the group expanded its scope to include low‐volume adult ARV products, and was renamed the ARV Procurement Working Group (APWG).

The theory of change underpinning the APWG is that coordinated procurement, where orders are consolidated with predictable ordering schedules, reduces lead times and avoids stock‐outs. Coordination occurs at the global level between member procurers, who in turn coordinate with their client programmes to negotiate acceptability of any adjustments. Sharing market intelligence across large funders and buyers ensures both visibility and confidence for manufacturers and supplier accountability. Supply disruptions are minimized and ARV markets are shepherded towards optimal formulations that benefit patients the most. The APWG consists of major funders and buyers like the Global Fund to Fight AIDS, Tuberculosis and Malaria, PEPFAR, UNICEF, national procurement units from Kenya and Ethiopia, and partners like UNITAID and CHAI. (It does not currently include South Africa procurement units.) Its approach includes:

1. Consolidated ordering at set times each quarter to ensure any one product has sufficient orders to fulfil a supplier's minimum batch size (ranging from 5000 to 50,000 packs). A quarterly review of planned procurements identifies potential issues around sub‐batch size and extended lead times are flagged early for corrective action. Buying plans can be adjusted while allowing members to adhere to their respective organizational policies.
2. Optimizing product selection using the formulary list developed by the IATT 19, and WHO guidelines on ART 14.
3. Aggregating a rolling quarterly forecast across procurers of demand by delivery quarter for the next 12 to 18 months to help suppliers with market visibility and production planning.
4. Regular structured dialogue between buyers, programmes and manufacturers to ensure timeliness and consistency of information sharing**.**
5. Collaboration with procurement partners to support improvement of country paediatric forecasting, procurement practices and supply management.

The APWG, responsible for well over half the global demand, has made great strides in stabilizing and streamlining the paediatric ARV market by consolidating volumes: lead times have reduced sharply, there is less fragmentation in product selection, and in 2016 less than 5% of orders by volume were “non‐essential” formulations as defined by the IATT. The APWG is an important body for downstream efforts to ensure that the new paediatric products are not only developed, but also realize their full potential in improving the lives of CLHIV, consistent with the targets of SDG 3.

### 2.4 Implementing the GAP‐f

The GAP‐f will ensure coordination among partners working in different areas in the paediatric field to achieve faster development and uptake of the most needed drugs for children. Implementation of the GAP‐f is conceptualized as a 3‐stage process:

1. Stage 1: Strategic development, consensus on activities to accelerate paediatric drug optimization and formalization of partner and stakeholder engagement.
2. Stage 2: Testing acceleration model for feasibility and results.
3. Stage 3: Launch of the GAP‐f as a fully functioning, sustainable structure informed by the evaluations of Stages 1 and 2.

The first stage will promote more visibility on the future market of individual priority products and regulatory efficiencies through increased coordination of the PIP/PSP processes in the European Union (EU) and the US:

1. Development of a harmonized master protocol for paediatric clinical, bioequivalence and palatability studies;
2. Increased engagement in high‐burden countries towards prioritizing registration of PADO priority products for children; and
3. Strategic assessment of timelines and durability of priority products (as prioritized by PADO).

In Stage 2, GAP‐f will build on the work of the PHTI to test its model for feasibility and results. This will include facilitation of early, effective engagement between innovators and paediatric HIV clinical trials networks to collaborate on the design of initial paediatric studies. Innovative approaches to incentivize generic development of priority products and promotion of earlier collaboration between innovators and generic manufacturers so that the generics can potentially be part of innovators’ development team and perform early child‐friendly formulation development will be considered. In collaboration with country partners, GAP‐f will develop harmonized messaging to ensure future market demand for priority products, simplified guidance for product introduction and product scale‐up plans.

In its full genesis as an independent entity (Stage 3), the GAP‐f will sustain and support activities and interventions proven to be effective in its initial stages. The experiences and lessons learned will inform the design of a fully functioning structure, which will coordinate and facilitate upstream and downstream activities detailed above. Evaluation of the impact of Stage 1 coordination efforts and promotion of more efficient drug trial designs in children and of Stage 2 examples of innovative financing and facilitated innovator‐generic manufacturer collaboration in paediatric formulation development will be incorporated into the final design of Stage 3 of GAP‐f. The GAP‐f mechanism will build on the HIV experience and subsequently expand its scope to include paediatric formulations of drugs for other critical disease areas, such as tuberculosis and viral hepatitis, which present a number of similar challenges.

## 3 Conclusions

The experience gained in paediatric HIV showed that the current separate work streams for development and uptake of paediatric formulations fall short to deliver optimal formulations for children and that a more structured and efficient collaboration is required. The GAP‐f proposes an innovative collaborative model, endorsed by key stakeholders, which will allow accelerated availability of safe, effective, quality‐assured and affordable paediatric medicines. It is essential to achieve the Start Free, Stay Free, AIDS Free super‐fast‐track targets of ending AIDS in children and adolescents by 2020 and will contribute to SDG 3 of reducing child mortality, ending AIDS and tuberculosis, and combating hepatitis. In addition, the GAP‐f represents an example of international access to innovation and effective public‐private partnerships, thus contributing also to SDG 17. The GAP‐f represents an example of extensive collaboration built on existing initiatives and partners’ expertise, with an ambitious overarching goal of accelerating access to best treatment options for diseases affecting children primarily in LMICs, such as HIV, tuberculosis, viral hepatitis and other infectious (and non‐communicable) diseases subject to similar market failures.

## Competing interests

MP, LL, MW, VP, FP, MA, WK, SM, MV, JL, DJ and GS have no competing interests to declare.

## Authors’ contributions

All authors contributed equally to drafting the content, editing and reviewing the manuscript for final endorsement.

## Funding

Part of this work (SM, MV) was supported by the International AIDS Society's Collaborative Initiative for Paediatric HIV Education and Research which is made possible through funding from founding sponsor ViiV Healthcare and from Janssen, and Industry Liaison Forum, through contributions from its 2016 and 2017 Gold Partners (Gilead Sciences, MSD and ViiV Healthcare), Silver Partners (AbbVie, Alere and Janssen) and Bronze Partners (Abbott, Beckman Coulter, bioLytical Laboratories, Cepheid, Cipla, Female Health Company, Lupin Pharmaceuticals, Omega Diagnostics, Roche Molecular Systems and Sysmex Corporation). Technical expertise for this document was supported in part (GS) by the U.S. President's Emergency Plan for AIDS Relief (PEPFAR).
